# Analysis of Nicotine Toxicity and Mechanisms of Senescence in Nucleus Pulposus Cells Using Network Toxicology and Molecular Docking Technique

**DOI:** 10.1002/jsp2.70055

**Published:** 2025-03-31

**Authors:** Chen Jiang, Chao Song, Chaoqi Chen, Baoxin Shen, Lei Yang, Chi Zhang, Fei Liu, Xiaofei Wu, Feng Chen

**Affiliations:** ^1^ Medical Insurance Section RuiKang Hospital Affiliated to Guangxi University of Chinese Medicine Nanning Guangxi China; ^2^ Department of Orthopedics RuiKang Hospital Affiliated to Guangxi University of Chinese Medicine Nanning Guangxi China

**Keywords:** cellular senescence, intervertebral disc degeneration, network toxicology, nicotine, protein acetylation

## Abstract

**Aim:**

Through the use of network toxicology, the research sought to determine whether cellular senescence and associated molecular mechanisms in nicotine‐induced intervertebral disc degeneration (IVDD) were potentially harmful.

**Methods:**

The primary chemical structure and 105 targets of action of nicotine were determined by using the Swiss Target Prediction, Cell Age, and PubChem databases. 855 IVDD senescence genes were found using the GEO and Cell Age datasets.

**Results:**

After additional screening and Cytoscape development, 9 key targets were identified. Additionally, these targets' co‐expression pattern analysis and protein interactions were confirmed to be identical. The core targets of nicotine‐induced IVDD cellular senescence were found to be primarily enriched in the positive regulation of cell proliferation, telomere shortening, histone acetylation, and cellular senescence‐related processes, according to gene ontology (GO) and Kyoto Encyclopedia of Genes and Genomes (KEGG). The KEGG signaling pathway also made it clear that the Apelin signaling route, nicotinate and nicotinamide metabolism, cell cycle, and apoptosis are all strongly linked to nicotine‐induced IVDD cellular senescence. We chose four genes associated with the cellular senescence pathway—HDAC1, HDAC4, and NAMPT, MYLK—for molecular docking with the toxic substance nicotine. The findings validated nicotine's strong affinity for the primary targets.

**Conclusion:**

All things considered, the current research indicates that nicotine may contribute to cellular senescence in IVDD via controlling the histone deacetylation process, telomere shortening, the Apelin signaling pathway, and pathways linked to the metabolism of nicotinate and nicotinamide. The theoretical foundation for investigating the molecular mechanisms of nicotine‐induced senescence in IVDD is established.

## Graphical abstract

The current study improves cyber toxicology as a method of evaluating environmental toxicity and molecular biological qualities in addition to clarifying the possible molecular pathways of nicotine‐induced senescence in IVDD.

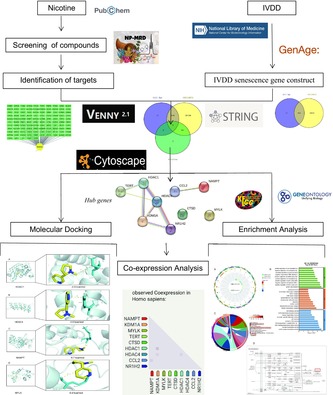

## 1 Introduction

Smoking is the leading cause of numerous illnesses, and tobacco smoke contains hundreds of dangerous chemicals that can affect practically every organ in the body [1]. A “common” risk factor for diabetes mellitus, cancerous tumors, cardiovascular and cerebrovascular disorders, and several chronic respiratory conditions is smoking [1, 2]. Over the course of this century, tobacco use may cause one billion fatalities globally, the most of which will take place in low‐ and middle‐income nations like China [3, 4]. With 40% of the world's population living there, China is currently the biggest tobacco consumer in the world. In China, men make up the great majority of smokers, with over two‐thirds of them smoking. Over a million Chinese people lose their lives to smoking‐related illnesses every year, and the numbers are still growing [5, 6]. According to epidemiology, smoking is closely linked to intervertebral disc degeneration (IVDD). According to certain research, smokers had an magnetic resonance imaging disc degeneration score that is roughly 20% greater than that of non‐smokers [7]. In another study, which followed nearly 60 000 teenagers for 11 years, smoking raised the likelihood of lumbar discectomy [8]. According to other research, smoking prolongs the healing process following disc surgery and aggravates pre‐existing disc degeneration [9, 10]. Despite this epidemiologic correlation, it is still unknown how smoking contributes to IVDD.

Smoking is a direct risk factor for accelerated disc aging and degeneration [11, 12]. Chronic coughing raises intra‐abdominal and intervertebral disc pressure, which increases the risk of disc herniation. Long‐term smokers also frequently experience respiratory disorders and more frequent coughing due to airway inflammation [13]. Nicotine in smoke will not only directly damage the human intervertebral disc but also cause blood vessel constriction, which lowers the intervertebral disc's nutrient supply and causes intervertebral disc aging and degeneration. Initially, the human intervertebral disc had less blood circulation of the tissue [14]. Smoking causes the creation of carboxyhemoglobin, which vasoconstricts blood arteries, reduces their lumen, and lowers blood flow by blocking oxygen transport in plasma [15]. Increased blood viscosity, which prevents oxygen transmission, atherosclerosis, which thickens the artery wall and lowers blood flow, and decreased fibrinolytic activity, which lessens the transvascular transport of nutrients to the disc [16]. The elasticity and resilience of the intervertebral disc, which is generally thought of as a flexible cushion that sits between the spine's bones, are crucial for preserving the spine's stability. The flexibility and durability of this cushion are further diminished by smoking, increasing the likelihood of herniated discs and making it more prone to damage [17]. Smoking weakens the muscles that support the spine and increases the risk of disc herniation by affecting muscular strength and endurance. Furthermore, weaker muscles will put more strain on the spine and hasten the discs' tendency to degenerate [18]. Nicotine, a primary component of tobacco smoke, has been demonstrated in vitro to decrease cell proliferation and the manufacture of extracellular matrix components in intervertebral disc cell cultures in a dose‐dependent way [19, 20]. Acute smoking dramatically lowers oxygen and glucose levels in the nucleus pulposus (NP) and constricts peridisc capillaries, according to experimental research conducted in a pig animal model [21]. When combined, these findings imply that smoking may contribute to IVDD, a key contributing factor to IVDD [21].

As an organism ages, its tissues and organs experience irreversible functional decline, a steady propensity toward death, a deterioration of the physiological stress response, and a weakening of the homeostatic regulation of the internal environment [22, 23]. Senescent cells play a key role in the development of IVDD. Through a number of different methods, senescent cells and senescence‐associated secretory phenotypes (SASPs) impact the local NP tissue, which ultimately results in an imbalance in the homeostasis of the intervertebral disc matrix. Biomarkers for cellular senescence include retinoblastoma protein (Rb), p38, p53, cell cycle protein‐dependent kinase inhibitors (p16 and p21), telomere length, and senescence‐associated β‐galactosidase (SA‐β‐gal) [24, 25]. A complex process of apoptosis and senescence causes IVDD. As the disease worsens, senescent cells—particularly senescent nucleus pulposus cells (NPCs)—accumulate within the IVD, and the severity of IVDD is positively connected with the quantity of senescent cells [25, 26, 27]. Apart from telomere shortening, DNA damage, and oxidative stress, cellular senescence can also be caused by abnormal mechanical loading, dysregulation of pro‐inflammatory factors, and disc cellular senescence. Recent research has also revealed that nicotine, a key ingredient in cigarettes, plays a role in the onset of cellular senescence [28, 29, 30]. According to a recent study, smoking cessation did not reverse the effects of two months of cigarette smoke exposure on disc structure, composition, and solute diffusion properties, especially in the cartilage endplate interface, in an SD rat model [28]. Researchers in another investigation on the function of DNA damage in smoking‐induced spinal degeneration discovered that short‐term exposure to high levels of primary tobacco smoke inhalation accelerated the degeneration of intervertebral discs and vertebrae. However, degeneration did not worsen in animals with homologous DNA repair deficiencies, indicating that DNA damage alone does not significantly contribute to smoke‐induced spinal degeneration [30]. Fewer studies have examined whether nicotine can cause cellular senescence in cellular IVDD, and more research is required to determine the precise molecular pathways underlying this phenomenon. However, there are some studies that link nicotine to IVDD and cellular damage.

**FIGURE 1:**
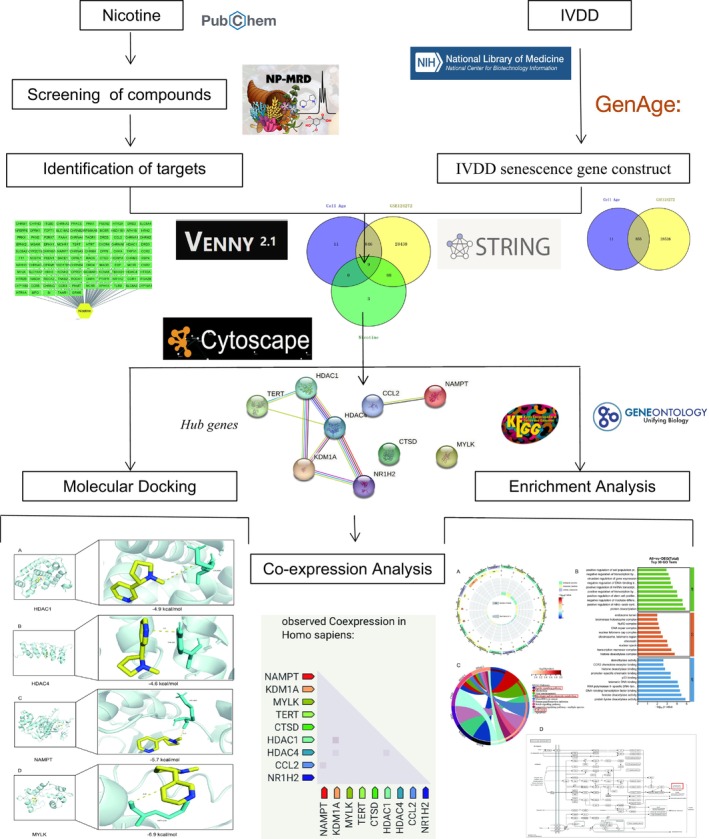
Flowchart of online toxicology of nicotine‐induced disc degeneration.

Network modeling has been widely employed since the introduction of the idea of network biology to investigate the rules and interactions among complex biological systems. Network toxicology is the process of creating particular network models to illustrate the toxicological characteristics of the research object and using network profiling to forecast a drug's toxicity to comprehend the harmful effects the drug has on the body and to forecast the toxic components and mechanisms of action of the drug [31]. Yizhi Zhang et al. investigated the toxicological mechanisms of IVDD caused by polyethylene terephthalate microplastics (PET‐MPs) using network toxicology and molecular docking techniques. This work highlights the value of network toxicology in determining the toxicity of newly discovered environmental contaminants and offers insightful information on the molecular mechanisms of IVDD caused by PET‐MPs [32]. Consequently, we think that research utilizing molecular docking and network toxicology can also help clarify the toxicological molecular pathways of nicotine‐induced cellular senescence in IVDD (Figure 1).

## 2 Materials and Methods

### 2.1 Nicotine Toxicological Information and Target Network Construction

A database of chemical modules is called Organic Small Molecule Bioactivity Data, or PubChem (https://pubchem.ncbi.nlm.nih.gov/) [33]. An overview of the physical and chemical characteristics of numerous molecular materials can be found on PubChem. To locate chemical and physical qualities, biological activity, safety and toxicity information, and more, we can search for chemicals using their names, molecular formulas, structures, and other identifiers. We used the chemical name of nicotine, the primary dangerous ingredient in cigarettes, to find pertinent toxicological data in PubChem for this investigation. NP‐MRD (https://np‐mrd.org/) is a database of NMR spectra of common compounds. Getting Nicotine's Spectral Information in NP‐MRD [34]. Swiss Target Prediction (http://swisstargetprediction.ch/), a compound target prediction tool, predicts the target of a compound based on the similarity of the 2D and 3D structures of known compounds [35]. Nicotine SMILES acquired using PubChem were utilized to anticipate potentially hazardous nicotine targets and build 2D chemical structures at Swiss Target Prediction. Cytoscape 3.10.0 was used to create nicotine‐target network diagrams.

### 2.2 Construction of Disc Degeneration Senescence Genes

Senescence genes can be downloaded from the Cell Age Database of Cell Senescence Genes (https://genomics.senescence.info/cells/), a database of genes comprising cellular senescence processes. Additionally, the GEO (https://www.ncbi.nlm.nih.gov/geo/) database, a public repository for gene expression data, was used to gather microarray data on disc degeneration GSE124272 [36]. Senescence gene data for the disc degeneration process was constructed by intersection analysis of microarray data and senescence genes.

### 2.3 Construction of Targets for Nicotine‐Induced Cellular Senescence During Disc Degeneration

To determine the targets of nicotine that cause the disc degeneration cells to senesce, the projected targets of nicotine were acquired by intersecting them with the data of senescence genes of the disc degeneration process. Based on data from public databases and literature, the STRING (https://cn.string‐db.org/) database is a networked database of protein interactions [37]. It gathers information from several public sources, combines it, and creates an extensive database of protein interactions. Click “SEARCH” on the site after opening the STRING database. To analyze multi‐protein interactions, choose “Multipleproteins” in the new interface. To move on to the next stage of creating a target network for nicotine‐induced cellular senescence in disc degeneration, enter or upload a list of gene or protein names and select human species under “Organization.” Then, click “SEARCH.” Interactions between the targets were discovered using co‐expression analysis of the hub genes in the STRING database. Differential violin plots were utilized to further screen for statistically significant genes for further investigation to evaluate the pathogenicity of the hub genes in the intervertebral discs.

### 2.4 Bioprocess Analysis of Core Targets

We employed the DAVID database for gene ontology (GO) analysis and enrichment of Kyoto Encyclopedia of Genes and Genomes (KEGG) pathways to investigate the biological roles of putative targets in nicotine‐induced aging. To clarify important biological activities, biological flux analyses addressed biological processes (BP), cellular components (CC), and molecular functions (MF) [38, 39, 40]. Furthermore, to find noteworthy pathways linked to possible targets of nicotine‐induced disc degenerative aging, KEGG pathway enrichment analysis was carried out. To improve the interpretation and presentation of the GO and KEGG analysis results, visualization analysis was finally carried out. We used the analysis software of BioBean (Shen‐Xin‐Dou‐Ya‐Cai) (http://www.sxdyc.com/) to chart the data.

### 2.5 Molecular Docking

The prior analysis yielded the predicted nicotine targets; however, additional verification is required to confirm this prediction. For this, the molecular docking technique can be employed to first show the compounds' capacity to bind to the target proteins and, consequently, their mode of action. The Pubchem database provided the suggested docking target's three‐dimensional structure in mol2 format. AutodockTools was then used to open, process, and store the tiny ligand molecules as pdbqt files. The RCSB protein database (www.rcsb.org/) offers the target protein's core 3D structure for download as a docking protein. After AutodockTools processing, save as a pdbqt file. The coordinates and box size of the Vina molecule for docking were determined by setting the parameter exhaustiveness to 15 and other parameters to default values. Autodock Vina 1.1.2 was used for semi‐flexible docking, and the best affinity conformation was selected as the final docked conformation [41].

### 2.6 Statistical Analysis

Plots and statistical analysis were carried out with Graphpad Prism 9.0. Histograms of mean ± standard error (SEM) values were used to display data from three or more separate studies. If the samples had a normal distribution, the two groups were compared using a *t*‐test; if not, a non‐parametric test was employed. Samples from several groups were compared using a one‐way Analysis of Variance [42]. Any two groups were compared using Tukey's method test, where a difference of *p* < 0.05 was considered statistically significant, and a difference of *p* < 0.01 was considered statistically significant.

## 3 Results

### 3.1 Toxicological Information on Nicotine and Results of the Pathogenic Target Network

**FIGURE 2:**
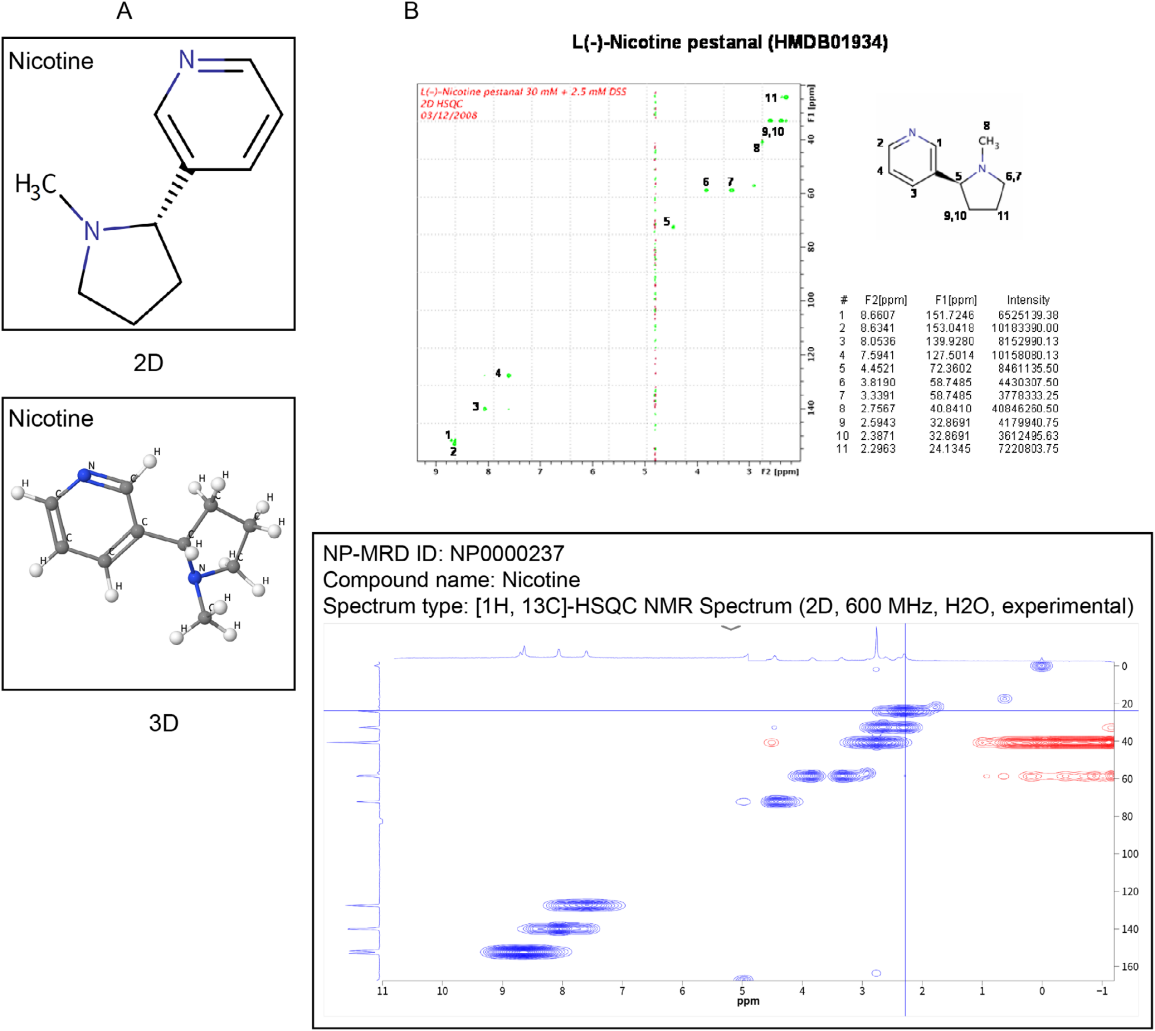
(A) 2D and 3D structural maps of nicotine. (B) online data spectra of nicotine.

**FIGURE 3:**
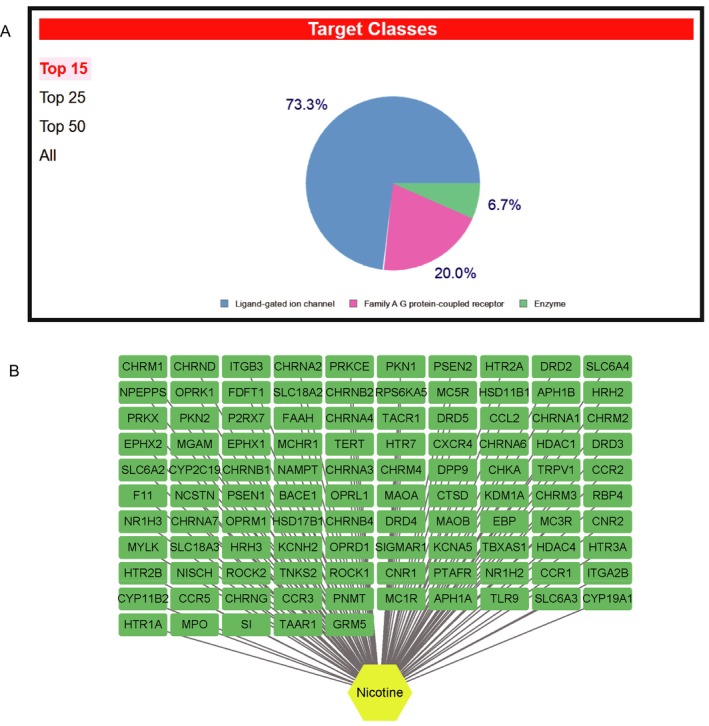
(A) Categorized pie chart of predicted targets of nicotine. (B) network diagram of predicted targets of nicotine.

We acquired toxicological data on nicotine, the primary dangerous ingredient in cigarettes, from the PubChem database. One of the main alkaloids in tobacco is nicotine, which is present in high concentrations in tobacco plants. Burning nicotine releases harmful nitrogen oxides. Nicotine is a colorless to pale yellow or brown liquid that is absorbed through the skin and respiratory system. Figure 2A displays its 2D and 3D structure. The spectral infographic displays the findings of the nicotine chemical tests that have been conducted thus far (Figure 2B). The 105 targets of nicotine's action that we ultimately identified were primarily involved in the regulation of ligand‐gated ion channels, family A G protein‐coupled receptors, and enzymes, according to target‐based categorization data (Table S1, Figure 3A,B).

### 3.2 Nicotine Results in Cellular Senescence During Disc Degeneration

**FIGURE 4:**
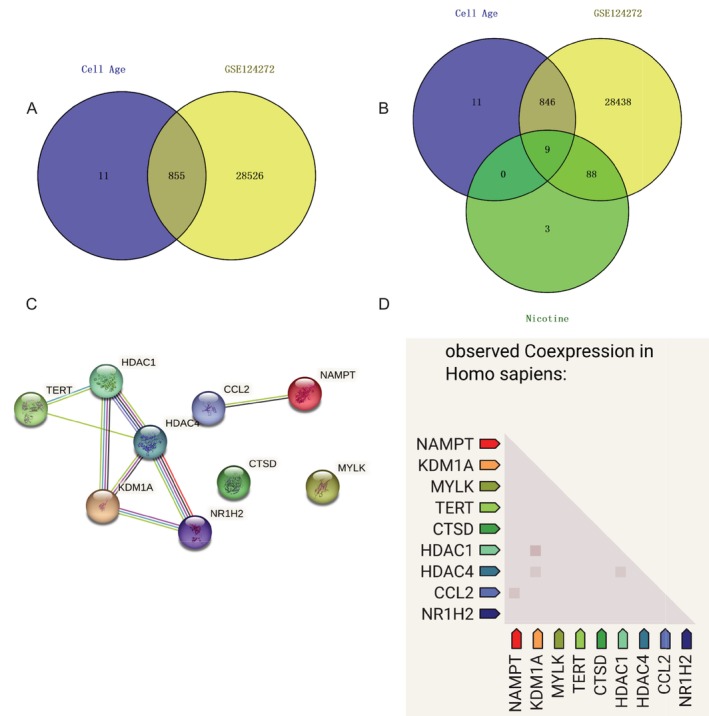
(A) Intersection of aging genes and disc degeneration data GSE124272. (B) Intersection of nicotine with disc aging genes. (C) Nicotine leads to intervertebral disc aging hub gene protein interactions. (D) Nicotine leads to intervertebral disc aging hub gene protein co‐expression.

We acquired 949 senescence genes from the cellular senescence gene database Cell Age (Table S2). We obtained the gene data GSE124272 for IVDD from the GEO microarray database. Sixteen blood samples—8 for IVDD and 8 for normal control—are included in this data set. Whole blood specimens from humans were collected for transcriptome sequencing in this study, and patient‐specific diagnostic information and patient addresses are shown in Table S3. After processing, 29 381 disease‐related genes for IVDD were found in the raw matrix that was ready for analysis (Table S3). Eight hundred and fifty‐five IVDD senescence process genes were obtained by intersecting senescence genes in the GSE124272 gene matrix (Figure 4A). Nine causative genes of nicotine that cause the senescence process of IVDD cells were obtained by taking the projected target genes of nicotine in the senescence process genes of IVDD and allowing them to intersect again (Table S4). We conducted interactions and co‐expression analyses between targets in the STRING database to better understand the relationship between these nine crucial genes. According to the findings of the protein contacts network, two proteins, CTSD and MYLK, were separated from other proteins, and there were protein interactions between CCL2 and NAMPT, HDAC1, HDAC4, KDM1A, NR1H2, and TERT. However, the findings of protein co‐expression indicated that there was gene co‐expression between HDAC1 and HDAC4, HDAC1 and KDM1A, NAMPT and CCL2, and HDAC1 and HDAC4 (Figure 4C,D). The two findings supported one another, suggesting that gluten‐induced IVDD cell senescence may be more heavily influenced by HDAC1, HDAC4, NAMPT, CCL2, and KDM1 A.

### 3.3 Results of Targeting Validation of Hub Genes

**FIGURE 5:**
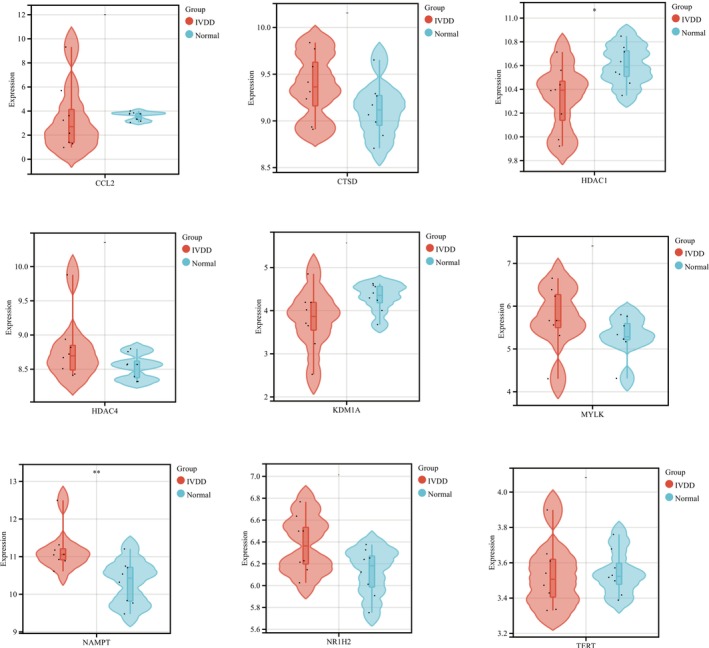
Differential box line plot of nicotine causing intervertebral disc aging hub genes.

We retrieved the gene expression in various tissue samples from the original matrix of GSE124272 and used difference box‐and‐line plots for statistical analysis to better understand how the nine pivotal genes impact the disease during IVDD. Nine genes were found to be different between sick and healthy participants. IVDD patients exhibited a trend of low expression of CTSD, HDAC4, MYLK, NAMPT, NR1H2, and TERT, and a trend of high expression of CCL2, HDAC1, and KDM1A. Statistical significance was achieved by NAMPT (** < 0.01) and HDAC1 (** < 0.05) among them (Table S5, Figure 5).

### 3.4 Results of Bioinformatic Process Analysis

The biological regulatory processes of the nine hub genes are primarily involved in metabolism, human diseases, organ systems, environmental information processing, and genetic information processing, according to the data analysis and presentation results of the online cloud platform.

**FIGURE 6:**
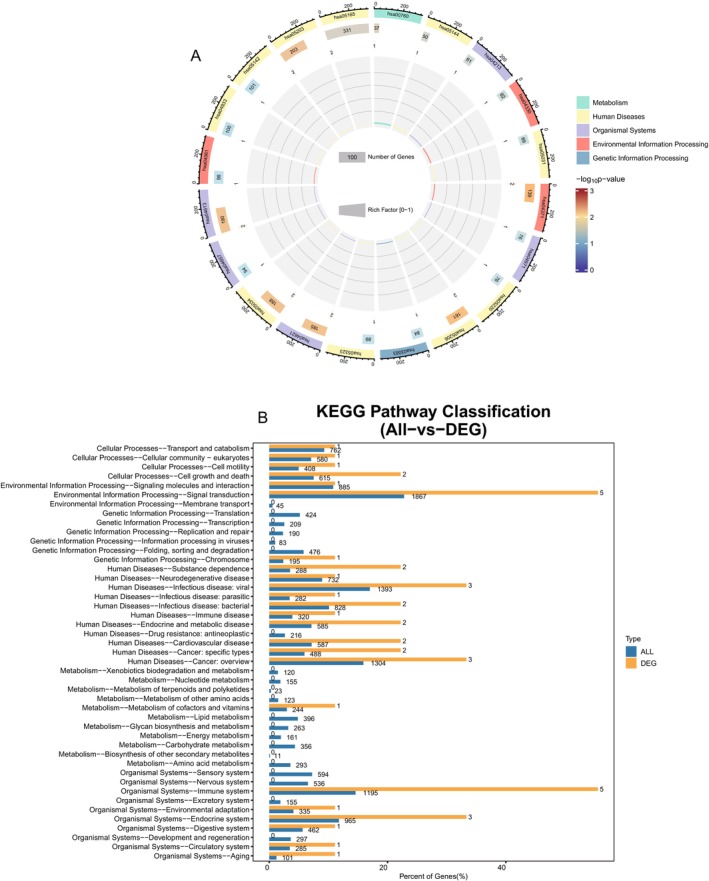
(A) Chord diagram of signaling pathway for hub gene enrichment analysis. (B) annotated bar graph of signaling pathway for hub gene enrichment analysis.

**FIGURE 7:**
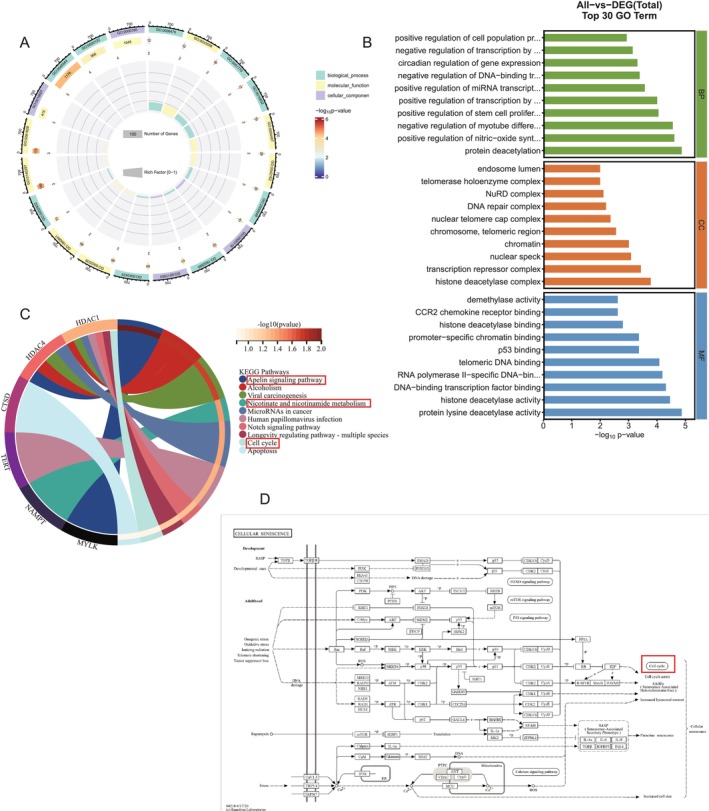
(A) Chord diagram of hub gene GO enrichment analysis. (B) Bar graph of hub gene GO enrichment analysis. (C) Chord diagram of hub gene KEGG enrichment analysis. (D) Senescence signaling pathway.

**FIGURE 8:**
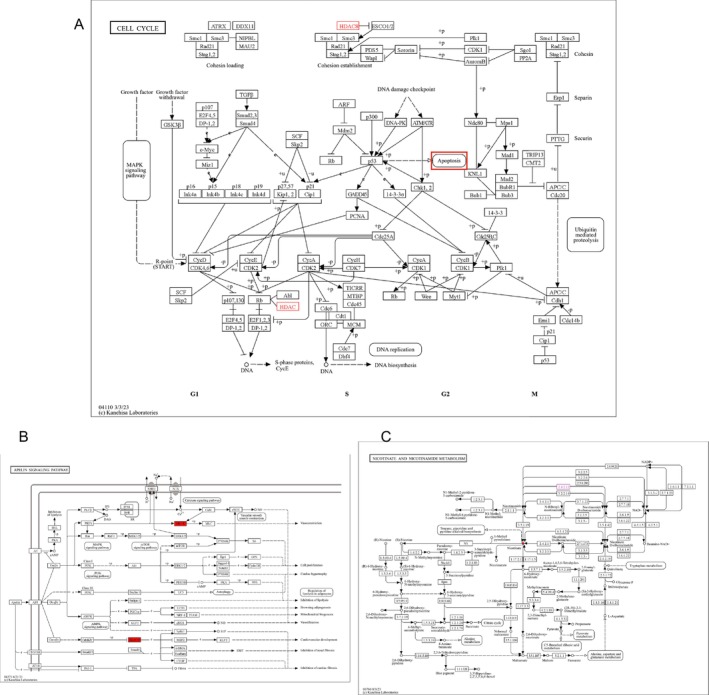
(A) Cell cycle signaling and key proteins. (B) Apelin signaling pathway and key proteins. (C) Nicotinate and nicotinamide metabolism and key proteins.

Signal transduction is a key process in environmental information processing; cellular processes focus primarily on cell growth and death; human diseases primarily include bacterial and viral infectious diseases; cancers: a summary; and organismal systems primarily focus on the immune and endocrine systems (Figure 6A,B). A total of 289 information entries were found using GO enrichment analysis (Table S6). Positive control over protein deacetylation and nitric oxide synthase biosynthesis, negative control over myotube differentiation, positive control over stem cell proliferation, positive control over RNA polymerase II transcription, positive control over miRNA transcription, negative control over dna‐binding transcription factor activity, circadian control over gene expression, negative control over RNA polymerase II transcription, and positive control over cell population proliferation are the primary components of BP regulation, the proliferation of cell populations is positively regulated. Information on nuclear speckles, chromatin, chromosomes, nuclear telomere regions, nuclear telomere cap complex, DNA repair complex, NuRD complex, telomerase holoenzyme complex, histone deacetylase complex, transcription repressor complex, and endosomal lumen are the primary entries covered for CC. DNA binding transcription factor binding, RNA polymerase ii‐specific DNA binding transcription factor binding, telomeric DNA binding, p53 binding, promoter‐specific chromatin binding, histone deacetylase binding, protein lysine deacetylase activity, histone deacetylase activity, and CCR2 chemokine receptor binding demethylase activity are the primary entries for MF. In conclusion, the mechanisms of protein deacetylation and telomeric DNA binding are intimately linked to cellular senescence during nicotine‐induced IVDD (Figure 7A,B). The Apelin signaling pathway, alcoholism, viral carcinogenesis, nicotinate and nicotinamide metabolism, microRNAs in cancer, human papillomavirus infection, Notch signaling pathway, longevity regulating pathway‐multiple species, cell cycle, and apoptosis were the primary signaling pathways that were impacted by the 57 relevant signaling pathways that were found to be expressed by KEGG enrichment analysis (Table S7, Figure 7C). We examined and filtered the main pathways from the KEGG database to further determine the pertinent pathways and the regulatory mechanisms of the proteins. First of all, it was proposed that the cell cycle pathway was a downstream expression signal in the cellular senescence pathway (Figure 7D). The cell cycle pathway had HDAC proteins involved, which also cause apoptosis, whereas MYLK and HDAC were primarily involved in controlling the Apelin signaling pathway. Finally, NAMPT target controlled the metabolism of nicotinate and nicotinamide as a signaling pathway linked to nicotine metabolism (Figure 8A–C). In conclusion, the signaling pathways for nicotinate and nicotinamide metabolism, the cell cycle, cellular senescence, and Apelin are all regulated by HDAC1, HDAC4, NAMPT, and MYLK.

### 3.5 Results of Molecular Docking

**FIGURE 9:**
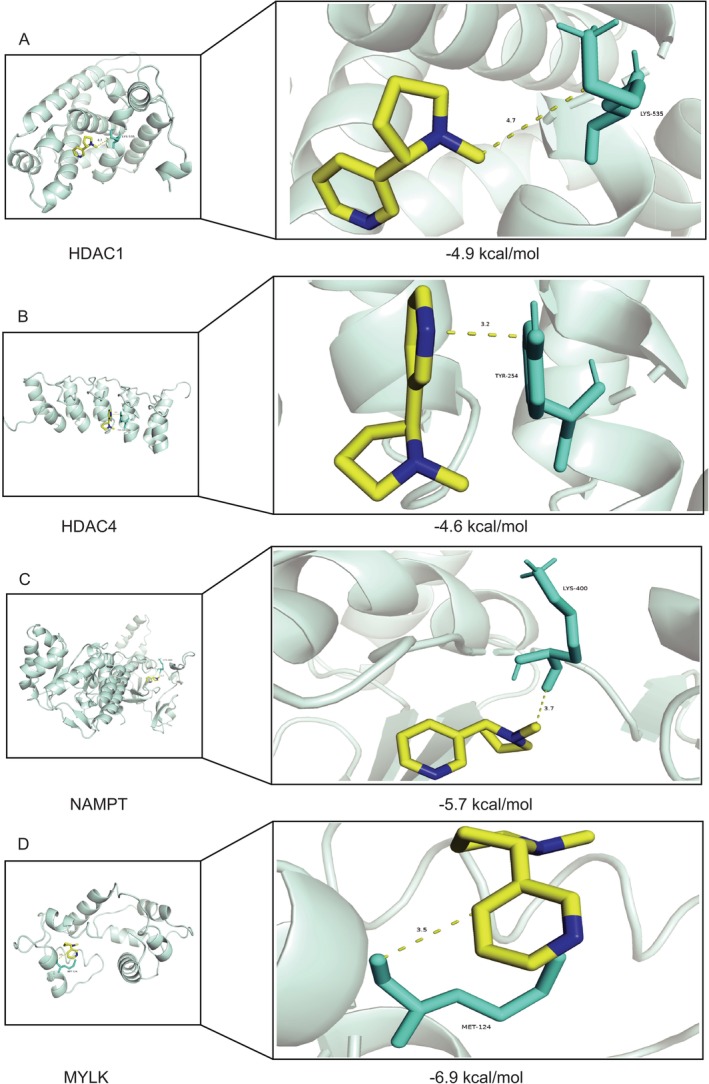
(A) Molecular docking pattern diagram of nicotine and HDAC1. (B) Molecular docking pattern diagram of nicotine and HDAC4. (C) Molecular docking pattern diagram of nicotine and NAMPT. (D) Molecular docking pattern diagram of nicotine and MYLK.

Four genes—HDAC1, HDAC4, NAMPT, and MYLK—were determined to be the hub genes for nicotine‐induced cellular senescence during IVDD based on bioinformatic process analysis. Using molecular docking research, we looked into how nicotine interacted with the four main target genes in the cellular senescence process. Nicotine's binding threshold for HDAC1, HDAC4, NAMPT, and MYLK was determined to be −4.9 kcal/mol, −4.6 kcal/mol, −5.7 kcal/mol, and −6.9 kcal/mol, respectively (Figure 9). In summary, nicotine can interact with the four senescence genes to have harmful consequences, according to the molecular docking studies, genes that can cause disease.

## 4 Discussion

IVDD is the most common cause of whiplash and low back pain, and although modern therapies can help, they cannot reverse the aging process [43]. The intervertebral disc is mostly reliant on the food supply from vertebral capillaries that penetrate the endplates because it is a nonvascular tissue with insufficient blood supply. According to earlier research, degenerative disc disease may arise as a result of an insufficient blood supply. Furthermore, epidemiologic and clinical research has clarified the risk factors for IVDD, including hyperlipidemia, alcoholism, and smoking [44]. Currently, 31.06% of Chinese adults over the age of 18 smoke, with adult males smoking at a rate of 59.7% and females smoking at a rate of 3.8% [45]. Smoking has detrimental effects in many orthopedic instances, such as nonhealing bone grafts in spinal fusion surgery, decreased bone mineral density, and delayed fracture healing [45]. Nevertheless, the impact of smoking on IVDD has not been well studied. Smoking's contribution to IVDD and potential senescence processes remain unclear. To further complement clinical epidemiologic studies and early research on the association between smoking and IVDD, this study sought to explore the mechanisms of smoking and cellular senescence during IVDD.

Using PubChem and other databases, we initially evaluated the targets of nicotine, the primary toxicant in cigarettes, for this investigation. Next, using the GEO, or cellular senescence database, we built the data of senescence genes in the IVDD process. We identified nine pathogenic genes through the intersection analysis of senescence genes and nicotine action targets in the process of IVDD. CCL2, HDAC1, and KDM1A showed a trend of high expression in IVDD patients, while CTSD, HDAC4, MYLK, NAMPT, NR1H2, and TERT showed a trend of low expression. HDAC1 and NAMPT were statistically significant among them. Hub gene‐based enrichment analysis showed that these genes primarily control telomere DNA binding and the protein deacetylation process. However, KEGG enrichment analysis revealed that the Apelin signaling pathway, the metabolism of nicotine and nicotinamide, the cell cycle, apoptosis, and other processes are strongly linked to nicotine‐induced cellular senescence in IVDD cells.

Gene expression is significantly influenced by post‐translational modifications of proteins, such as acetylation and ubiquitination. Histone acetylation, in particular, is mostly found at lysine residues and has a role in controlling biological processes and gene expression. To maintain protein stability and transcriptional activity, histone acetyltransferases (HAT) and histone deacetylases (HDAC) control the acetylation and deacetylation, respectively, of non‐histone proteins, including transcription factors. The acetyl group is eliminated from histones and non‐histone proteins by the HDAC enzyme family. They can be categorized into three groups based on their structure and homology: class I (HDAC1‐3 and HDAC8), class II (HDAC4‐10), and class III (SIRT1‐7) [46]. Deterioration of biological processes may result from senescence, which is linked to a reduction in DNA replication, protein synthesis, and cell division activities. It was discovered that overexpression of HDAC1 also improved the interaction between Sp1 and p300 and raised the degree of deacetylation of Sp1. In the end, our work showed that HDAC1 overexpression, via a unique Sp1/PP2A/pRb pathway, reduced proliferation and caused cervical cancer cells to undergo premature senescence [47]. Another study discovered that HDAC4 deficiency causes senescence, the buildup of damaged DNA by drawing BRCA1 and CtIP to the lesion site, and H2BK120ac accumulation. In summary, the buildup of damaged DNA and the activation of transcriptional programs regulated by super‐enhancers that sustain senescence are caused by the breakdown of HDAC4 during senescence [48]. Cheng Zheng et al. observed that by enhancing mitochondrial activity, HDAC/H3K27ac‐mediated NDUFA3 transcription protected human myeloid cells challenged with high glucose. The primary molecular processes include oxidative phosphorylation by HDAC, reduction of ROS, enhancement of mitochondrial function, and prevention of apoptosis. Furthermore, a number of studies have demonstrated the tight relationship between cellular senescence in the process of IVDD and ROS‐mediated mitochondrial malfunction and NPC apoptosis [49]. Thus, based on previous research as well as the current work, we may conclude that nicotine contributes to the histone deacetylation process via HDAC1 and HDAC1, which causes ROS buildup, mitochondrial damage, and death in NPCs, finally resulting in NPC senescence.

In the mammalian nicotinamide adenine dinucleotide (NAD) repair route, nicotinamide phosphoribosyltransferase (NAMPT) is a rate‐limiting enzyme that is essential for controlling cellular metabolic activity, reprogramming, senescence, and death [50]. Through the nicotinate and nicotinamide metabolism pathway, NAMPT enzymatically synthesizes nicotinamide mononucleotide (NMN), a crucial protein implicated in host defense mechanisms and crucial for maintaining metabolic balance and cell survival [50, 51]. According to theory, one of the causes of the age‐related drop in NAD+ levels is the reduction in NAD+ synthesis. This condition is believed to be primarily caused by decreasing enzymatic activity and NAMPT protein levels. Chronic inflammation brought on by other cellular stressors, such as oxidative stress and environmental stressors like smoking, may also be the source of the age‐related decrease in NAMPT [52]. It was discovered that NAMPT contributes to the matrix degradation brought on by IL‐1β, and NLRP3 inflammatory vesicles are essential for IVDD. Huang et al. discovered that by suppressing the activity of NLRP3 and NAMPT inflammatory vesicles, melatonin might reduce the matrix degradation caused by TNF‐α. Furthermore, NAMPT inhibited NLRP3 inflammatory vesicle activity to regulate TNF‐α‐induced matrix degradation by downregulating MAPK and NF‐κB signaling in NPCs [53]. Another study discovered that by administering exogenous NAMPT to revitalize senescent NPCs and endplate cells, tiny extracellular vesicles made from adipocytes could reduce disc degeneration in mice [54]. Consequently, we think that nicotine stimulation following cigarette inhalation causes NLRP3 inflammatory vesicle activation, which exacerbates matrix breakdown and causes NPC and endplate cell senescence. Although the precise molecular mechanisms behind these activities need to be further confirmed, they are strongly linked to the control of nicotine in NAMPT, as well as the metabolism of nicotinate and nicotinamide.

An essential signaling molecule in the adipocytokine signaling route is apelin, which is also a part of the apelin signaling pathway. The apelin signaling route is a multipurpose signaling mechanism that is involved in energy metabolism, inflammation, and the cardiovascular system in both normal and pathological ways. According to recent research, the Apelin/APJ system is crucial to aging. The expression of Apelin and APJ receptors decreases with aging [55]. While Apelin decrease results in increased vigor as well as behavioral and circadian characteristics that restore vigor, Apelin and APJ knockouts in mouse models show accelerated senescence [56]. Through the AMPK/SIRT1 signaling pathway, the apelin/APJ axis mitigates AngII‐induced vascular endothelial cell senescence; the underlying mechanism may be linked to increased telomerase activity and decreased ROS generation [57]. Further research has revealed that the Apelin‐13/APJ system delays disc degeneration by activating the PI3K/AKT signaling pathway, promoting proliferation, reducing inflammation and apoptosis, and reducing the breakdown of the extracellular matrix of the NP [58]. Studies on the Apelin signaling system, which controls cellular senescence during IVDD, are scarce, nonetheless. However, according to a recent study, aging made aortic aneurysm entrapment worse by activating the miR‐1204‐MYLK signaling axis PEVuZE5vdGU [59]. Senescence‐induced miR‐1204 inhibits myosin light chain kinase (MYLK) and encourages the development of a SASP, which results in the production of cytokines and chemokines and vascular inflammation. One of the proteins that controls the Apelin signaling pathway is MYLK. As a result, these investigations further suggest that MYLK triggers the release of secretory phenotypes linked to senescence via the Apelin signaling pathway, which could be a significant contributing factor to nicotine‐induced senescence in NPCs.

This study used molecular docking and network pharmacology analysis to quickly evaluate the possible toxicity of these protein‐acetylated compounds while also clarifying the molecular pathways of nicotine‐induced aging and its possible toxicity. Potential targets and chemical signaling pathways can be found without the need for drawn‐out animal testing, in contrast to typical toxicological investigations. The effectiveness of screening and evaluating the toxicity of chemical substances is greatly increased when network toxicology and molecular docking approaches are used. The study will help people to understand the dangers of smoking and thus lead people to quit smoking. At the same time, it is instructive to develop blocking drugs for diagnostic targets based on the target effect of toxic substances. This may have implications for future withdrawal treatment in severely addictive patients in the clinic. It is crucial to remember that this study still has certain issues. First, the analysis's trustworthiness and data volume are constrained because it is based on a single, small sample of GSE124272. Second, the pertinent targets of the current investigation have not been the subject of many direct experimental studies, both domestically and internationally. We think that the current study is nonetheless important in spite of its flaws.

## 5 Conclusion

We discovered that nicotine contributes to multiple targets and multiple signaling pathways that result in the process of total cellular senescence in IVVD. NAMPT, MYLK, HDAC1, and HDAC4 are the main targets. We essentially clarified the molecular mechanisms by which nicotine causes cellular senescence in IVDD. First, nicotine regulates NAMPT and the metabolism of nicotinate and nicotinamide, which are engaged in the senescence process of NPCs, and it also plays a role in the histone deacetylation process through HDAC1, which results in apoptosis and NPC senescence. Nicotine may potentially trigger the release of secretory characteristics linked to aging by mediating the Apelin signaling pathway via MYLK. The current study improves cyber toxicology as a method of evaluating environmental toxicity and molecular biological qualities.

## Author Contributions

**Chao Song:** data analysis, Writing – original draft. **Chen Jiang:** data analysis, Writing – original draft. **Feng Chen:** funding acquisition, providing technical support. **Chi Zhang:** image analysis. **Xiaofei Wu:** image analysis. **Chaoqi Chen** and **Baoxin Shen:** performing the experiments. **Lei Yang:** conceptualization, methodology, supervision, funding acquisition. All authors participated in this article.

## Ethics Statement

The authors have nothing to report.

## Consent

The authors have nothing to report.

## Conflicts of Interest

The authors declare no conflicts of interest.

## Supporting information


**Table S1.** Predicted targets of nicotine.
**Table S2.** Cell senescence gene.
**Table S3.** GSE124272 matrix data.
**Table S4.** The intersection genes of nicotine causing disc aging.
**Table S5.** Expression levels of intersection genes where nicotine causes disc senescence.
**Table S6.** GO enrichment analysis of the intersection genes where nicotine causes disc senescence.
**Table S7.** KEGG enrichment analysis of the intersection genes for nicotine‐causing disc senescence.

## Data Availability

All data are in the manuscript and/or Supporting Information files.
